# Therapeutic Potential of Alpha-pinene in Breast Cancer: Targeting miR-21 and PTEN Gene Expression

**DOI:** 10.31661/gmj.v13i.3613

**Published:** 2024-11-30

**Authors:** Mohammad Yazdi, Mahdi Alaee, Shana Ahadi, Parisa Khanicherag, Elham Ghayem, Ghasem Farhadi Galil Babadi, Fatemeh Ghiasi, Mojgan Azadpour

**Affiliations:** ^1^ Razi Herbal Medicines Research Center, Lorestan University of Medical Sciences, Khorramabad, Iran; ^2^ Department ofclinical Biochemistry, Faculty of Medicine, Lorestan University of Medical Sciences, Khorramabad, Iran; ^3^ Shahid Rajaee Hospital, Qazvin University of Medical Sciences, Qazvin, Iran; ^4^ School of Medicine, Jundishapur University of Medical Sciences, Ahvaz, Iran; ^5^ Student Research Committee,Tabriz University of Medical Sciences, Tabriz , Iran; ^6^ Department of clinical Biochemistry and Labratory Medicine, Tabriz University of Medical Sciences, Tabriz, Iran; ^7^ Department of Internal Medicine, School of Medicine, Ahvaz Jundishapur University of Medical Sciences, Ahvaz, Iran; ^8^ Instructor of critical care nursing, Department of Anesthesiology, School of Allied Medical Sciences, Ayatollah Taleghani Hospital, Ilam University of Medical Sciences, Ilam, Iran

**Keywords:** Breast Cancer, miR-21, PTEN, Alpha-pinene

## Abstract

Background: Alpha-pinene is an organic compound with anticancer properties. This compound has been used as a therapeutic factor in breast cancer (BC). miR-21 causes cancer cell invasion by inhibiting Phosphatase and tensin homolog (PTEN). The present study evaluates the potential effect of Alpha-piene on the expression of PTEN and miR-21 genes in BC cells (MCF-7 cell line).Materials and Methods: In this study, the MCF-7 cell line was used. The cells were treated with Alpha- pinene. The viability of cells with different Alpha-pinene concentrations (i.e., 40, 50, 100, 150, and 200 μM) was evaluated with MTT assay. The expression of PTEN and miR-21 genes was evaluated using RT-qPCR. Results: The survival rate of cells in all concentrations was higher 24 h after treatment compared to 48 h after the treatment (P0.0001). The expression of miR-21 in cells treated with 100 and 50 μMof Alpha- pinene reduced significantly compared to the control cells(P0.001). PTEN gene expression was exactly the opposite of miR-21. Therefore, its expression increased significantly in cells treated with 100 μM of Alpha- pinene compared to the control cells (P0.0001). Conclusion: In general, the use of Alpha- pinene led to decreased and increased expression of miR-21 and PTEN, respectively. These changes lead to the reduction of invasion and proliferation of BC cells. Therefore, the Alpha- pinene combination can be used as a therapeutic strategy to treat patients.

## Introduction

Breast cancer (BC) is one of the malignancies that most occurs in women. Based on the evidence, more than 2 million people with BC have been identified in the world (in 2018), and more than 600 thousand people have died [1][2][3]. The pathophysiology of BC is multifactorial; so, many factors are impressive in the occurrence of BC [4][5]. Environmental risk factors such as age, gender, economic status, and place of residence are among the elements that affect the incidence of BC. Based on the established evidence, genetic disorders have an important role in the occurrence of disease. Besides, dysfunction of genes and molecular pathways have significant effects on the occurrence and progression of BC [6][7][8][9]. As an organic compound, anticancer properties of Alpha-pinene have been identified to a large extent. Research has proven that this combination inhibits the proliferation and invasion of tumor cells. So far, rare studies have been conducted regarding the effect of Alpha-piene on BC cells [10].

Phosphatase and tensin homolog (PTEN) are two genes involved in DNA repair. When cells and genes are damaged, PTEN is activated and repairs the damaged genes [11]. Disruption in its function and structure leads to cancer. Evidence shows that PTEN is disrupted in many cancers, including BC. Disruption of PTEN prevents cell apoptosis [12][13]. miR-21 is an oncomir variant with an increasing expression in many cancers, including BC. Disruption of miR-21 expression leads to proliferation, prevention of apoptosis, and invasion of BC [14][15].

Previous studies confirmed the anticancer effects of Alpha-pinene. However, the effect of alpha-pinene on BC cells has been limited. miR-21 causes the proliferation of cancer cells by regulating the expression of PTEN. Although the expression of these two genes has been evaluated in BC cells, the effect of Alpha-pinene on PTEN and miR-21 genes in BC cells has not been evaluated. Therefore, we used the MCF-7 cells as BC cells in this study.

## Materials and Methods

**Figure-1:**
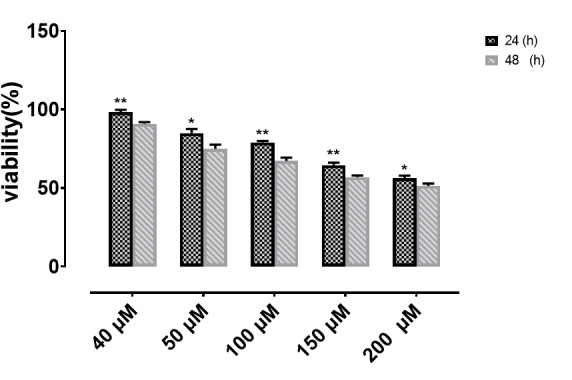
Figure 1. The MTT assay related Alpha-pinene (40, 50, 100, 150, and 200 μM concentration); *<0.05, **<0.01.

Purchasing and Preparation of Cell Culture

The MCF-7 cell line was purchased from the Pasteur Institute, Tehran.

The cells were cultured in a medium supplemented with some components including 12% fetal bovine serum (FBS) and 1% penicillin-streptomycin. After culture, the cells were incubated in the incubator at 37° with 5% CO2. The subculturing process was done every 3-4 days.

MTT Assay

Typically, the 3-(4,5-dimethylthiazol-2-yl)-2,5-diphenyltetrazolium bromide (MTT) test is used to evaluate the viability and proliferation of cells after incubation with a specific substance [16]. To perform this test, first, a certain number of MCF-7 cells (100 μM containing 104 cells per well) was transferred into 96-well plates. After 24 hours, specific concentrations of Alpha-pinene (Merck company, Germany) (40, 50, 100, 150, and 200 μM) were added to each well. The experiments were conducted in triplicate. The two 96-well plates were incubated at 37° with 5% CO2 and 98% humidity. Following a 24-h and 48-h incubation period, respectively, 100 μM of serum-free media and 10 μM of MTT solution (final concentration 0.5 mg/mL) were added to each well. The plate was then incubated at 37° for a further 3 h. After this incubation period, 150 μMof MTT solvent was added to each well. The plate was then wrapped and incubated for a further 15 min. Subsequently, the optical absorption of each sample was determined at a wavelength of 570 nm using an ELISA reader.

RNA Extraction

RNA extraction from cells was performed using the relevant kit (Jena Bioscience, Germany) and following the kit instructions. The quantity and quality of the extracted RNA were evaluated by NanoDrop and electrophoresis, respectively. In the next step, cDNA was synthesized using the kit (Jena Bioscience, Germany) and according to its instructions.

Real-time PCR (RT-qPCR)

The expression of PTEN and miR-21 genes was evaluated using RT-qPCR. The Real-Time PCR System (Applied Biosystems, USA) was used to perform the PCR amplification under the following conditions: 10 min of initial denaturation at 95°C, 40 cycles of denaturation at 95°C for 15 s, and 1 min of annealing/extension at 60°C. Genes were normalized to the housekeeping gene of GAPDH and the expression was assessed using the 2-ΔΔCt method.

Statistical Analysis

Data analysis was conducted by SPSS software (Version 22, IBM Corp., Armonk, NY., USA)

. The t-test was used to compare gene expression in cells. Post hoc analysis was also used to determine the viability of Alpha-pinene on the MCF-7 cell line. P<0.05 was considered significant in all analyses.

## Results

**Figure-2:**
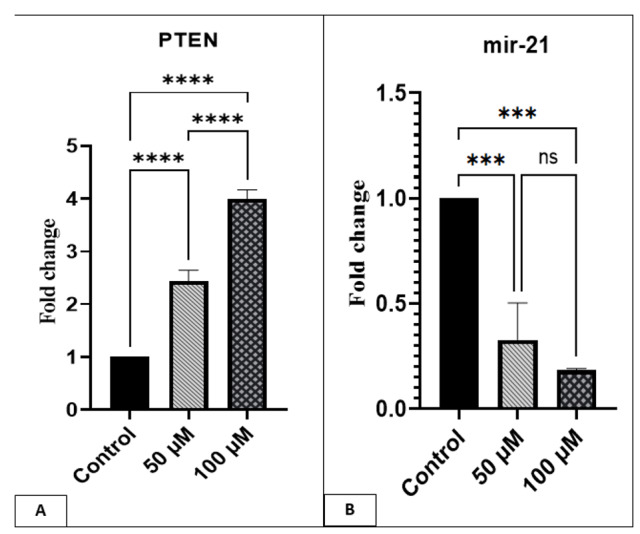
Figure 2. The expression of miR-21 and PTEN genes in cells treated with Alpha-pinene.

**Table:** Table 1. Post Hoc Analysis of Viability of MCF-7 Cell Line Treated with Alpha-pinene at 24 and 48 h After Exposures

Time (h)			Alpha-pinene concentrations			P-value
	40µM	50µM	100µM	150µM	200µM	
24h	98.34±1.53 ^a^	85.00**±**2.65 ^b^	79.00±1.00 ^c^	64.67±1.53 ^d^	56.34±1.53 ^e^	P<0.0001
48h	91.33±1.00 ^a^	75.00±2.65 ^b^	67.33±2.082 ^c^	57.00±1.00 ^d^	51.33±1.53 ^e^	P<0.0001

In general, the MCF-7 cells were first treated with Alpha-pinene, and the cell viability and toxicity were evaluated. In the next step, the expression of miR-21 and PTEN genes was evaluated in cells.

Cell Viability Assay

Figure-1 shows the viability of cells treated with different concentrations of alpha-pinene at 24 and 48 h after the treatment. The results at both 24 and 48 h indicated that the viability of cells at the same concentrations of alpha-pinene was significantly higher at 24 than 48 h post-treatment (P<0.0001).

The Table-1 revealed a significant difference between all the concentrations at 24 and 48 h.

Based on these results, the 50 μM and 100 μM dose were chosen as the substance concentration in subsequent experiments.

Gene Expression of miR-21 and PTEN

The expression of miR-21 in cells treated with 100 and 50 μM of Alpha-pinene was reduced compared to control cells (P<0.001). PTEN gene expression was exactly the opposite of miR-21; its expression increased in cells incubated with 100 and 50 μM of Alpha-pinene compared to the control cells (P<0.0001, Figure-2).

## Discussion

The expression of miR-21 in cells treated with 100 and 50 μM of Alpha- pinene were compared to the control cells (P<0.001). PTEN gene expression was exactly the opposite of miR-21; its expression increased significantly in cells treated with 100 and 50 μM of Alpha- pinene compared to the control cells (P<0.0001). Previous study that Alpha- pinene has an antagonist effect on cancer cells, thereby lowering their survival rate. Some studies have shown that Alpha-pinene inhibits the invasion of tumor cells by inducing apoptosis and reducing proliferation [10][17]. Kang et al. showed that Alpha-pinene inhibits the NF-κB and prevents the secretion of MMPs and VEGF by targeting the TNF-α. Inhibiting the production of these factors led to the prevention of angiogenesis and ultimately the inhibition of the proliferation of BC cells [10]. In another study, Ghanbariasad et al. showed that Alpha-pinene increased the expression ratio of BAX/BCL-2 in BC cells. This change led to the induction of apoptosis in BC cells [17].

According to the results, Alpha-pinene mainly affects the proliferation, inflammation, and apoptosis of cells. Accordingly, the caspase 3 and BCL-2 expression were increased and decreased, respectively. Also, the Alpha-pinene prevents inflammation by inhibiting the NF-kB pathway [18]. Furthermore, reducing the expression of Cdc25c and CDK1 lowers the proliferation of cells and stops the cell cycle [19].

Another noteworthy point is that miR-21 controls cell proliferation through the regulation of ERK/MAPK pathways. The activation of the ERK/MAPK pathway increases the expression of AKT, and finally BCL-2. It also causes the progression of the cell cycle by activating the mTOR [20][21].

In previous studies, miR-21 expression was shown to be altered in BC cells. Based on this, Nalinie et al. showed that the miR-21 increased the proliferation of BC cells. Target genes of miR-21 included PTEN, Pdcd4, and BCL-2 that involved in apoptosis. Reduce expression of miR-21 increases the expression of PTEN and down-regulates the Pdcd4 and BCL-2, leading to apoptosis of BC [22].

Gong et al. showed that the use of antisense oligonucleotides to inhibit miR-21 increases the expression of PTEN. It was also found that increased expression of PTEN leads to the sensitivity of BCs to Trastuzumab [23]. In another study, Qian et al. reported that miR-21 acts as an oncogene. The increase in its expression leads to the activation of TGF-β [24]. Increased expression of miR-21 increases drug resistance in cancer patients, especially BC [25]. In this regard, Wang et al. showed that the use of curcumin in patients leads to an increase in the sensitivity of cells to chemotherapy drugs. Additional investigations showed that curcumin leads to the reduction of miR-21 expression, thereby increasing PTEN expression and preventing AKT phosphorylation. Finally, these signaling pathways lead to the reduction of BCL-2 expression and induction of apoptosis in cells [26]. On the other hand, it has been found that the expression of miR-21 in the cell invasion of BC is higher compared to the non-invasive cells, which indicates the role of this miR in cell invasion [27].

The present study has some limitations. First, genes related to cell proliferation and apoptosis were not investigated. Second, it would also be better to perform a protein assay to increase the validity of the results. Third, the signaling pathways related to miR-21 and PTEN genes were not evaluated.

## Conclusion

Overall, the use of Alpha-pinene reduces the expression of miR-21 and increases the expression of the PTEN gene, thereby lowering the invasion and proliferation of BC cells. Therefore, the Alpha-pinene combination can be used as a therapeutic strategy to treat patients. In other words, Alpha- pinene along with other treatments may improve clinical symptoms and treat patients.

## Conflict of Interest

None.
